# Developmental Interplay between Ethnic, National, and Personal Identity in Immigrant Adolescents

**DOI:** 10.1007/s10964-021-01434-y

**Published:** 2021-04-17

**Authors:** Stefanos Mastrotheodoros, Olga Kornienko, Adriana Umaña-Taylor, Frosso Motti-Stefanidi

**Affiliations:** 1 Department of Youth and Family, Utrecht University, Utrecht, The Netherlands; 2 Department of Psychology, George Mason University, Fairfax, GA USA; 3 Harvard Graduate School of Education, Harvard University, Cambridge, MA USA; 4 Department of Psychology, University of Athens, Athens, Greece

**Keywords:** Immigrant adolescent, Ethnic identity, National identity, Personal identity, Longitudinal

## Abstract

Developing a personal identity is a core developmental task for all adolescents. Immigrant adolescents need to integrate the meaning that their belonging to their ethnic group and the receiving nation has for them into their personal identity. The purpose of this study was to examine the longitudinal interplay between personal, ethnic, and national identities of a middle school sample of immigrant youth (*N* = 765, *M*_age_ = 12.7 years, *SD* = 0.6 at T1; 46% girls) enroled in Greek schools. Data were collected in three waves with repeated measures. To test the link between these identities, two trivariate Cross-Lagged Panel Models were ran, one examining identity exploration and the other examining identity commitment. The results revealed robust within time positive links between ethnic, national and personal identities for both exploration and commitment at all three time-points. There was some evidence that ethnic and national identities were negatively linked longitudinally, and limited support for longitudinal associations between these domains and personal identity. Follow-up analyses suggest that these processes may be specific to second generation youth and that findings may differ by ethnic background. Finally, the findings that emerged are discussed with attention to the socio-political climate in the receiving nation.

## Introduction

The formation of an identity involves different aspects of self-definition and, while being challenging for all adolescents, it is even more challenging for immigrant adolescents, who have to navigate between (at least) two different cultures: the culture of the country of origin and the culture of the host country (Motti-Stefanidi et al., 2012). Just as their non-immigrant peers, immigrant adolescents need to explore and define themselves with respect to various personal identity domains (e.g., education, interpersonal relationships; Crocetti et al., 2008). Additionally, immigrant adolescents also need to explore and understand the meaning their ethnicity has for them, ideally integrating into their self-definition characteristics of the culture of origin (ethnic identity) with characteristics of the host culture (national identity), in which they live and develop (see Nguyen & Benet-Martínez, 2013). Personal, ethnic, and national identities comprise distinct aspects of the broad construct of identity that have typically been addressed in separate literatures (Schwartz et al., 2011). Due to this divided literature, there is limited understanding regarding how these aspects of identity co-develop during adolescence. Given the global increasing rates of immigrant youth, this lack of knowledge is an important gap, as it hinders understanding of how immigrant youth develop their sense of self. The present study investigated the longitudinal links among processes of personal, ethnic, and national identities, in a sample of immigrant adolescents in Greece.

### Personal Identity

Adolescence is a challenging developmental stage as it brings with it accelerated development in areas such as cognition, emotion, self-regulation, parenting (Mastrotheodoros et al., 2019), family relationships (Branje et al., 2021), and peer relationships. Young people are expected to gradually address different developmental tasks, of which developing a personal identity is especially challenging, because it requires that young people explore and synthesize answers related to multiple life domains such as education, and interpersonal relationships. Specifically, these two identity domains revolve around key developmental tasks, and have been shown to have important implications for adolescent adjustment and psychological well-being, a finding replicated across cultural groups (Dimitrova et al., 2018). In addition, because of their importance, these two identity domains have been used to approximate personal identity development in studies investigating personal and social identities (Albarello et al., 2018).

Developmental scholars have focused on understanding two processes underlying the formation of personal identity. *Exploration* refers to the active questioning and weighing of alternative roles, beliefs, values, and life plans, before deciding which to adopt and pursue. *Commitment making* involves personal investment in particular alternatives, and the adoption of a course of action that will lead to the implementation of these choices (Kroger & Marcia, 2011). These dimensions have been shown to differentially relate to adolescent psychological adaptation, with higher levels of personal identity commitment, and lower levels of personal identity exploration predicting better psychological outcomes (e.g., Schwartz et al., 2009).

### Ethnic and National Identity

People do not only define themselves in terms of their future and goals related to education and interpersonal relationships, but also in terms of their membership(s) in social groups (Tajfel & Turner, 2004). These aspects of self-definition refer to individuals’ social identities (Spears, 2011). Ethnic and national identities are aspects of social identity that refer to membership to, or association with, ethnic and/or national groups, respectively (Umaña-Taylor, 2011; Umaña-Taylor et al., 2014). *Ethnic identity* refers to a self-constructed internalization of a person’s group membership based on that person’s attitudes and feelings toward his/her cultural background, ethnic heritage, and racial phenotype, whereas *national identity* captures individuals’ ‘subjective or internalized sense of belonging to the nation’ (Umaña-Taylor et al., 2020).

Ethnic and national identity revolve around questions such as ‘what does a person’s ethnicity or connection to the nation where they reside mean to them?’ ‘How central is this social identity to their sense of self?’ (Umaña-Taylor et al., 2014). Ethnic and national identities are investigated in this study in terms of the *processes (exploration and commitment)* by which young people develop these identities, and not in terms of their *content* (Umaña-Taylor et al., 2014). Ethnic and national identity *exploration* captures their search for information about these social identities. In contrast, ethnic and national identity *commitment* concerns the understanding and acceptance of one’s ethnic background.

### Interplay of Personal, Ethnic, and National Identities

Several theoretical arguments support the concurrent and longitudinal links between personal, ethnic, and national identities. First, all three identities are aspects of the more general self-system (Oyserman et al., 2012). Also, all three identities are formed within the constrains and opportunities provided by the context οf the receiving society. Nonimmigrants may or may not have positive attitudes towards the presence of immigrants in the country, which may affect the formation of these identities for immigrant youth (Motti-Stefanidi, 2021). Therefore, changes in one of these identity domains are expected to correlate with, or even precipitate changes in the other identity domains. Second, at least some aspects of personal identity may be assigned, informed, or constrained by ethnic or national group membership (Schwartz et al., 2018), or cultural orientation (Triandis, 2001). Thus, personal identity might be affected by national and ethnic identities.

Despite these theoretically plausible links, only few empirical studies have longitudinally investigated whether and how personal, ethnic, and national identities inform each other as they unfold over time. A study conducted in the US with recently immigrated Latinx adolescents found that personal identity coherence longitudinally predicted increases in ethnic and national identity commitment, suggesting that understanding who they are and where they are going in life predicts over time better understanding of what their cultural background and their membership to the receiving nation means to them (Meca et al., 2017). Even though reciprocal effects were also documented, whereby ethnic and national identity commitment longitudinally predicted personal identity commitment, these effects were smaller. Another study also conducted in the US with a mixed sample of immigrant and non-immigrant college students found that current and past personal identity exploration were only weakly and positively (if at all) associated with ethnic identity exploration (Schwartz et al., 2009). Thus, evidence on the link between personal and ethnic identity exploration is inconclusive, whereas the link between personal and national identity exploration has not been examined.

Apart from the links between, on the one hand, personal identity and on the other hand, ethnic and national identity processes, the interplay between domains of social identity—ethnic and national identities—is also important to investigate further. For immigrant youth, forming ethnic and national identities are acculturative tasks (Phinney et al., 2001), whose outcome becomes part of the developmental task of forming a personal identity (Motti-Stefanidi, 2021). Forming an identity as ‘being both’ a member of one’s ethnic group and of the receiving nation is a key criterion of positive adaptation among immigrant youth (Wiley et al., 2019). Given that multiple identities are simultaneously explored and defined (Syed & McLean, 2016), theory and evidence (Martinez-Fuentes et al., 2020) suggest that developing identity in one domain may inspire or hinder identity development in other domains.

### The Role of Socio-Political Context

The relationship between developmental processes of immigrant adolescents’ personal identity domains as well as ethnic and national identities, depends to a large extent on the socio-political climate in the receiving nation (Ward & Geeraert, 2016). In assimilationist societies, immigrant youth are forced to choose between the national culture and their own culture, resulting in a negative relationship between the two. In contrast, in more receptive multicultural societies immigrant youth are given space to keep and nurture both cultures, resulting in a positive relationship between them.

Greece is a more assimilationist than receptive society toward immigrant people. According to an OECD (2016) report, 60% of Greek citizens indicate that they view immigrant people unfavourably, and more than two thirds of the Greek population believe that immigrant people do not contribute to the country’s collective well-being. According to a joint OECD and European Commission report (2015), 35% of the immigrant people in Greece report feeling discriminated against, which ranks Greece first in terms of immigrants’ perceived discrimination in Europe.

In Greece, like in other non-settler European societies that tend to be more assimilationist (Verkuyten et al., 2019), national orientation and commitment may be incompatible with ethnic orientation and commitment (Pavlopoulos & Motti-Stefanidi, 2017). Recent empirical evidence showed that perceived discrimination (Jugert et al., 2020) as well as country-level inequalities (e.g., gender or income inequalities) (Jugert et al., 2019), contributed to lower ethnic identification and/or a lower compatibility between national and European identities. Thus, in countries like Greece, with relatively assimilationist attitudes and apparent inequalities between non-immigrant and immigrant youth, negative associations between ethnic and national identity processes might be expected.

In sum, extant evidence on the interplay between personal, ethnic and national identities is sparse and is based on studies which have been conducted in the US, often using college students. However, the societal context in which young immigrants develop is expected to affect the link between their ethnic and national identities (Ward & Geeraert, 2016), as well as whether and how ethnic and national identities are linked to personal identity (Schwartz et al., 2018).

## Current Study

The present study aimed at investigating the longitudinal interplay between personal, ethnic, and national identity exploration and commitment via three research questions. First, it was examined whether and how the three identity domains were linked within time (Research Question 1). It was expected that they would be positively linked within time based on the arguments outlined earlier, and particularly on the argument that they are aspects of the more general self-system. Second, it was examined whether and how ethnic and national exploration and commitment were linked over time (Research Question 2). For both identity processes (exploration and commitment), they were expected to be negatively linked over time. Third, it was examined whether and how personal identity processes (exploration and commitment) were linked with ethnic and national identity processes, respectively, over time (Research Question 3). Regarding identity exploration, the analysis was exploratory since extant evidence has been sparse and inconclusive. Regarding identity commitment, it was expected that personal identity commitment would positively predict ethnic and national identity commitment.

## Methods

### Sample

The sample consisted of 765 immigrant early adolescents (*M*_age_ = 12.7 years, *SD* = 0.6 at T1; 46% girls) who participated annually in a longitudinal 3-wave study following students from Grade 7 to Grade 9, in public junior high schools in Athens, Greece. The schools were specifically targeted to include a high proportion of immigrants. Immigrant status was specified as having at least one parent born in a country other than Greece (adolescent-reported), a criterion that has been applied to other studies from the same research project (Motti-Stefanidi & Asendorpf, 2017). Most (*n* = 597, 78%) were second generation immigrants (students who were born in Greece), whereas the rest (*n* = 168, 22%) were born in a country other than Greece. First generation immigrants had spent on average 61% of their lifetime in Greece (range 1–99%). Most students were of Albanian decent (*n* = 382, 49.9%), the rest being Pontian-Greek (*n* = 149, 19.5%), and ‘other’ (*n* = 234, 30.6%). Socioeconomic status was conceptualized as level of socioeconomic adversity, and it was a composite score based on several indicators, such as mothers’ and fathers’ education and profession, living in own housing (vs paying rent), and housing density (number of people living under the same roof divided by the number of rooms available). The adversity score ranged from 0 to 4 (higher scores indicating higher socioeconomic adversity), and this sample showed a low-moderate adversity at T1 (*M* = 1.3, *SD* = 0.9).

Participation rate was high, as most students (*n* = 563, 73.6%) participated in all three waves, many (*n* = 136, 17.8%) participated in two waves, and only a minority (*n* = 66, 8.6%) participated in only one wave. To test for differences between students who participated in all three waves and students who did not, t-test comparisons were conducted on all study’s variables (demographics, personal, ethnic, and national identity exploration and commitment). As seen in Table S1 (in the supplementary material), only four out of 24 tests were significant. Students who participated in all three waves had lower socioeconomic adversity at Waves 2 and 3 (*p*’s = 0.001, and 0.029, Cohen’s *d* = 0.36 and 0.33, respectively), higher personal identity exploration at T3 (*p* = 0.029, Cohen’s *d* = 0.33) and were younger on average at T1 (*p* < 0.001, Cohen’s *d* = 0.81) than students who missed at least one wave.

### Measures

#### Ethnic identity and national identity

Ethnic Identity was measured with the Revised Multigroup Ethnic Identity Measure (Phinney & Ong, 2007), a 6-item scale that assesses the degree to which students engage with and feel connected to their ethnic culture. To assess National Identity, students were asked to respond to the same 6 items with reference to the Greek culture. Therefore, the same items are used to assess both national and ethnic identity; only the reference culture (origin vs Greek) differs in the two forms of the scale. Across both ethnic and national identity scales, the items comprised two subscales capturing developmental dimensions: Exploration (e.g., ‘I have spent time trying to find out more about my ethnic group, such as its history, traditions, and customs’, ‘I have often talked to other people in order to learn more about my ethnic group’), and Commitment (e.g., ‘I have a strong sense of belonging to my own ethnic group’, ‘I feel a strong attachment towards my own ethnic group’). Participants responded to each item using a 4-point scale ranging from 1 (*completely disagree*) to 4 (*completely agree*). Two composites were computed for ethnic and national identity: exploration, and commitment. The two subscales showed acceptable internal consistency coefficients *α* for both ethnic identity (ranging from 0.76–0.87 for exploration and commitment across waves) and for national identity (ranging from 0.64–0.83 for exploration and commitment across waves, see Table 1).

**Table 1:** Means, standard deviations, bivariate correlation coefficients and cronbach’s alpha for the dimensions of personal, ethnic and national identity

Variable	*M*	SD	1	2	3	4	5	6	7	8	9	10	11	12	13	14	15	16	17	18
1. Pers_Com. 1	3.82	0.72																		
2. Pers_Exp. 1	3.58	0.68	0.58**																	
3. Ethn_Com. 1	2.93	0.89	0.22**	0.18**																
4. Ethn_Exp. 1	2.81	0.87	0.23**	0.26**	0.71**															
5. Nat_Com. 1	2.87	0.90	0.22**	0.27**	0.04	0.10*														
6. Nat_Exp. 1	2.65	0.81	0.26**	0.30**	0.10*	0.27**	0.66**													
7. Pers_Com. 2	3.49	0.64	0.37**	0.41**	0.09	0.13**	0.19**	0.22**												
8. Pers_Exp. 2	3.37	0.72	0.25**	0.43**	0.06	0.11*	0.19**	0.20**	0.89**											
9. Ethn_Com. 2	2.84	0.88	0.12**	0.14**	0.38**	0.35**	−0.05	0.02	0.19**	0.16**										
10. Ethn_Exp. 2	2.65	0.81	0.19**	0.17**	0.32**	0.41**	−0.05	0.10*	0.27**	0.20**	0.73**									
11. Nat_Com. 2	2.82	0.89	0.08	0.09*	−0.03	0.03	0.54**	0.39**	0.22**	0.21**	0.01	0.02								
12. Nat_Exp. 2	2.57	0.81	0.13**	0.16**	−0.04	0.08	0.42**	0.42**	0.28**	0.24**	0.10*	0.26**	0.67**							
13. Pers_Com. 3	3.48	0.70	0.34**	0.20**	0.10*	0.07	0.08	0.11*	0.50**	0.35**	0.11*	0.15**	0.14**	0.22**						
14. Pers_Exp. 3	3.27	0.67	0.17**	0.38**	0.02	0.10*	0.15**	0.17**	0.49**	0.54**	0.07	0.08	0.17**	0.23**	0.51**					
15. Ethn_Com. 3	2.84	0.89	−0.00	0.06	0.33**	0.34**	−0.11*	0.02	0.10*	0.07	0.42**	0.38**	−0.03	0.02	0.17**	0.11*				
16. Ethn_Exp. 3	2.63	0.84	0.04	0.09	0.28**	0.33**	−0.11*	0.02	0.17**	0.13**	0.36**	0.41**	−0.07	0.04	0.23**	0.21**	0.72**			
17. Nat_Com. 3	2.79	0.85	0.06	0.07	−0.05	0.00	0.42**	0.30**	0.19**	0.15**	−0.00	0.03	0.53**	0.42**	0.17**	0.17**	0.09*	0.11**		
18. Nat_Exp. 3	2.52	0.78	0.12**	0.12*	−0.03	0.07	0.30**	0.34**	0.25**	0.21**	0.03	0.13**	0.40**	0.43**	0.21**	0.21**	0.12**	0.29**	0.63**	
Cronbach’s α			0.82	0.73	0.84	0.77	0.80	0.64	0.83	0.78	0.83	0.76	0.83	0.71	0.83	0.77	0.87	0.82	0.82	0.68

*M* and SD are used to represent mean and standard deviation, respectively

*Pers_Com* Personal Identity Commitment, *Pers_Exp*: Personal Identity Exploration, *Ethn_Com* Ethnic Identity Commitment, *Ethn_Exp* Ethnic Identity Exploration, *Nat_Com* National Identity Commitment, *Nat_Exp* National Identity Exploration

*p < 0.05; **p < 0.01

#### Personal identity

Personal Identity was assessed with the Utrecht-Management of Identity Commitments Scale (Crocetti et al., 2008). Using 10 items, two identity dimensions are assessed: Identity Commitments and In-Depth Exploration. Furthermore, this scale assesses these dimensions in two different domains, namely Education and Friendship, using 10 similarly worded items for each domain. For this study, the two domains were collapsed to achieve a more robust measure of personal identity dimensions, following past studies using the same instrument and that conceptualized personal identity in this same way (Crocetti et al., 2017). Example items are: ‘My education/best friend gives me security in life’ (Identity Commitments), ‘I often try to find out what other people think about my education’ (In-depth Exploration). Participants responded to these items on a scale from 1 (*completely untrue*) to 5 (*completely true*). This scale has been translated and used in many languages and has been shown to be measurement invariant in many countries (Crocetti et al., 2015), including Greece (Mastrotheodoros et al., 2021). The internal consistency coefficients for the two dimensions were acceptable, ranging from 0.74 to 0.83 across scales and waves (see Table 1).

### Procedure

The study was approved by the Greek Ministry of Education. Data collection took part from 2013 to 2015. Trained research assistants visited middle schools in Athens metropolitan area. School principals were informed, parents were asked for informed consent, and students were asked for assent. Participants completed paper-and-pencil surveys during three school hours, spread over two visits in each school. The questionnaires were available in Greek, Albanian, Russian, and English, in case students preferred to address the items in a different language. All students opted to complete their survey in Greek language.

### Analytic Procedure

Before proceeding to the main analyses to answer the research questions three preliminary steps were followed. First, the sample was screened for outliers following the currently suggested best practices (Aguinis et al., 2013). These consist of techniques to detect univariate, multivariate, and prediction outliers, and they were applied separately for each wave and for each variable. Binary variables were computed that indicated whether a case was a univariate, multivariate, and/or predictor outlier in each of the waves and each of the variables. All binary variables were summed across waves and in the following analyses only those cases were used that had a sum outlier score less than 3. That is, only cases were used that were either not indicated to be outliers at all (sum score 0) or were found to be outlying only according to one way (univariate, multivariate, or predictor outlier) on only one or two waves in one variable, or one or two variables in one wave (sum score 2). This step resulted in the analytic sample of 723 students (exclusion of 42 students).

Second, using the *naniar* package in R (Tierney & Cook, 2018) the missing data patterns were visualized for all items that would be used as indicators in the latent variable models later. To control for attrition in these analyses, missingness was examined for the three waves separately, only among students who did participate in each wave. Missingness ranged from 6.6 to 18% at T1, from 9.2 to 15.4% at T2, and from 8.6 to 12.7% at T3. To handle missingness, multiple imputation was applied using the mice package in R (van Buuren & Groothuis-Oudshoorn, 2011), requesting 20 multiply imputed data sets over 10 iterations each.

As a third preliminary step, using the *semTools* package in R (Jorgensen et al., 2020), a series of measurement invariance analyses were performed to test whether the underlying meaning of the scales differed across time for the ethnic identity and national identity scales (MEIM), but not for the UMICS scale because longitudinal measurement invariance has already been shown (Mastrotheodoros et al., 2021). The semTools package uses algorithms that allow latent variable models estimation over multiply imputed data and pools the results. To test longitudinal measurement invariance, the items of both ethnic and national identity dimensions were specified as ordinal. Following recent guidelines for testing invariance of ordered categorical indicators (Wu & Estabrook, 2016) increasingly stricter models were specified to examine configural, thresholds, loadings, and intercepts invariance. These models test whether the items measure the same number of latent factors in the same way across the three timepoints of this study. Invariance was assumed when imposing the additional parameter constraints implied by each of these models did not lead to a large decrease in fit, indicated by changes of less than 0.015 in RMSEA and SRMR, and less than 0.010 in CFI (Cheung & Rensvold, 2002). Table 2 presents the results of the invariance analyses. Both scales showed invariance of factor structure, item factor loadings, item thresholds, and item intercepts, indicating that the meaning of the items, and their associations with the latent constructs remained comparable across time.

**Table 2:** Tests of longitudinal measurement invariance for the two-factor models of ethnic identity and national identity

	χ²	*df*	CFI	RMSEA [90% CI]	SRMR	Δ*χ*^2^ *p*	ΔCFI	ΔRMSEA	ΔSRMR
Ethnic identity									
Configural	58	102	1.00	0.000 [0.000–0.000]	0.033				
Thresholds	58	114	1.00	0.000 [0.000–0.000]	0.033	0.990	0.000	0.000	0.000
Loadings	60	122	1.00	0.000 [0.000–0.000]	0.033	0.682	0.000	0.000	0.000
Intercepts	68	130	1.00	0.000 [0.000–0.000]	0.033	0.021	0.000	0.000	0.000
National identity									
Configural	102	102	1.00	0.000 [0.000–0.020]	0.035				
Thresholds	97	114	1.00	0.000 [0.000–0.010]	0.035	0.998	0.000	0.000	0.000
Loadings	96	122	1.00	0.000 [0.000–0.000]	0.036	0.337	0.000	0.000	0.001
Intercepts	108	130	1.00	0.000 [0.000–0.006]	0.036	0.114	0.000	0.000	0.001

The Wu & Estabrook (2016) procedure for identification of ordinal indicators was applied in *semTools* 0.5–3 (Jorgensen et al., 2020). Pooled results based on 20 multiply imputed data sets, generated over 30 iterations each

*CFI* comparative fit index, *RMSEA* root mean square error of approximation, *SRMR* standardized root mean residual

The main analyses to answer the research questions were two trivariate autoregressive cross-lagged panel models: Model 1: Personal Identity Exploration, National Identity Exploration, and Ethnic Identity Exploration; and Model 2: Personal Identity Commitment, National Identity Commitment, and Ethnic Identity Commitment. A latent variable framework was used to account for the uncertainty in the measures. In the case of personal identity, given the large number of items, parcelling was performed by collapsing the domains of education and friendship, following exactly the same parcelling solution that has been used in many studies using the U-MICS (Crocetti et al., 2008). Because the indicators for both ethnic and national identity were on ordered categorical scale, the diagonally weighted least squares (DWLS) estimator was used. The *semTools* package in R (Jorgensen et al., 2020) was used to run the models using multiply-imputed data and to pool the results. In both models, control variables included: adolescent gender, ethnicity, immigrant generation, and family SES.

## Results

Descriptive statistics and correlations for all study variables are presented in Table 1.

### Interplay of Personal, National, and Ethnic Identity Exploration

All variables showed moderate to strong positive, significant autoregressive effects that ranged from *β* = 0.52 to *β* = 0.65. These effects indicated that personal, national, and ethnic identity exploration were stable across time, and those students who reported relatively high degree of exploration at one time point tended to also show high degree of exploration the following time points.

**Fig. 1:**
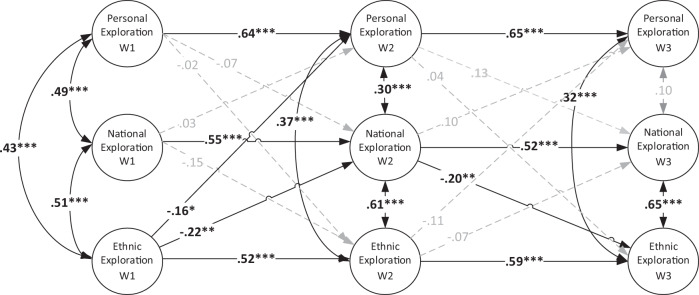
Standardized coefficients for the cross-lagged effects among Personal Identity Exploration, National Identity Exploration and Ethnic Identity Exploration. Adolescent sex, ethnicity, immigrant generation and family SES were controlled for. Results are pooled over 20 multiply imputed data sets, generated over 10 iterations each. *N* = 723. CFI = 1.00; TLI = 1.20; RMSEA = 0.000; *x*^2^ = 161, df = 441

The most prominent finding was that exploration in the personal, ethnic, and national identity domains is a positively correlated intertwined process (see Fig. 1). T1 correlations and all but one correlated residual at Waves 2 and 3 were positive. This indicates that, starting from early adolescence, immigrant adolescents who reported a relatively greater exploration in one domain of their identity (e.g., ethnic identity) tended to also report a relatively greater exploration in the other domains of identity (e.g., personal and national). The positively correlated residuals among most of the pairs in T2 and T3 indicated that, compared to other students, those who increased in exploration in one domain tended to also increase, relative to their classmates, in exploration in other domains.

Also, significant cross-lagged associations emerged. A negative longitudinal association between ethnic identity exploration and national identity exploration emerged from T1 to T2. Students who showed greater ethnic identity exploration at T1, relative to their classmates, tended to show relatively less exploration of their national (Greek) identity at T2. Also, a negative longitudinal association emerged between national identity exploration and ethnic identity exploration from T2 to T3. Students who showed higher national Greek identity exploration at T2, relative to classmates, tended to show a relative decrease in their ethnic identity exploration in T3. Finally, the model revealed a negative cross-lagged association from ethnic identity exploration at T1 to personal identity exploration at T2, indicating that those students who reported relatively higher exploration of their ethnic identity, compared to their classmates, over time tended to show relatively decreased exploration of personal identity aspects in T2. This pattern did not emerge from T2 to T3.

### Interplay of Personal, National, and Ethnic Identity Commitment

All variables showed moderate to strong positive, significant autoregressive effects that ranged from *β* = 0.42 to *β* = 0.60. These effects indicated that personal, national, and ethnic identity commitment were stable across time, and those students who showed relatively high commitment at one time point tended to also show relatively high commitment the following time points.

**Fig. 2:**
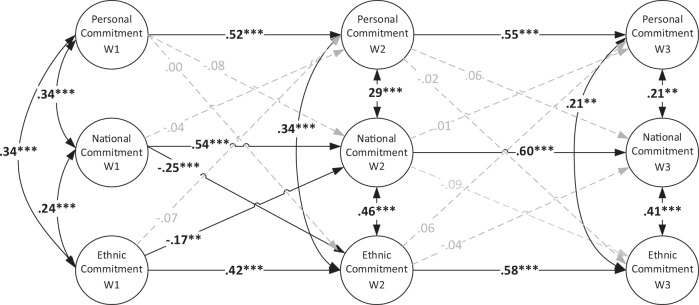
Standardized coefficients for the cross-lagged effects among Personal Identity Commitment, National Identity Commitment and Ethnic Identity Commitment. Adolescent sex, ethnicity, immigrant generation and family SES were controlled for. Results are pooled over 20 multiply imputed data sets, generated over 10 iterations each. *N* = 723. CFI = 1.00; TLI = 1.07; RMSEA = 0.000; *x*^2^ = 189, df = 441

Similar to exploration, the model for commitment showed a pattern of positive within-time associations, among personal, national, and ethnic identity (Fig. 2). This indicates that adolescents who, compared to their classmates, show stronger commitments in one domain of their identity (e.g., ethnic identity) tend to also show stronger commitments in the other domains of identity (e.g., personal, and national).

Also, a negative and reciprocal dynamic pattern emerged between national identity commitments and ethnic identity commitments, between T1 and T2. Those students who committed more to their national Greek identity during T1, compared to their classmates, were those who committed less to their ethnic identity during T2, and vice versa. In other words, on the group level, greater national identity commitments in early adolescence are linked with an ensuing decrease in ethnic identity commitments and, simultaneously, greater ethnic identity commitments in early adolescence are linked with a subsequent decrease in national identity commitments.

### Sensitivity Analyses

Immigrant generation and ethnicity were included as controls in the primary analyses. However, to explore potential moderation by these sociodemographic characteristics, sensitivity analyses were conducted using multigroup modelling. Specifically, two sets of multigroup analyses were conducted, one set for exploration and one for commitment (see Figs. S1–S4). The multigroup models tested one socio-demographic characteristic as the moderator (e.g., immigrant generation) while controlling for the second (e.g., ethnicity), and vice versa. Due to sample size limitations, testing both moderators simultaneously was not warranted. Overall, several of the patterns that emerged in the main findings were replicated but there was some evidence that the findings may apply largely to 2nd generation youth and that some associations may vary by ethnicity, although statistical power issues may have precluded some coefficients from reaching significance.

Regarding immigrant generation, for the most part, the coefficients were in the same direction and of similar size for 1st and 2nd generation immigrants. Two exceptions attract attention: in the case of exploration, the effect of T1 ethnic identity exploration on T2 personal identity exploration, as well as the effect of T1 national identity exploration on T2 ethnic identity exploration were positive (yet, nonsignificant) in the 1st generation immigrant group, but negative (and significant) in the 2nd generation group. As can be seen in Figs. S1 and S2, for the 1st generation immigrant group, fewer effects reached significance, which might be because of this group was underpowered (*N* = 152).

Regarding ethnicity, a similar picture emerged. For the most part, effects were of similar size and direction in all three groups (Albanian, Pontian Greek, other). Statistical power for the separate groups (e.g., only 138 Pontian Greek, and 223 Other) was low, and large coefficients failed to reach significance. In the case of exploration, the picture that emerged from the main analyses (negative longitudinal links between ethnic and national exploration) held mostly for Albanian and for Other. In the case of commitment, the negative longitudinal effect of T1 national commitment on T2 ethnic commitment held for all three groups, but the reverse only held for Albanians and Other. Two cross-lagged effects emerged in these analyses that did not emerge in the main analyses. For Pontian Greek, T2 national exploration and T2 ethnic exploration had, respectively, a positive and a negative effect on T3 personal exploration.

## Discussion

Identity development is a core developmental task during adolescence (Erikson, 1968) that has important implications for youth’s future adaptation. For immigrant adolescents, developing an identity might be especially challenging. Apart from the more universal aspects of identity in terms of exploration of and decisions over the domains of education and interpersonal relationships (conceptualized as personal identity in the current study), immigrant youth must also explore and commit to often conflicting aspects of social identity, like ethnic identity and national identity. Ethnic, national, and personal identities have most often been studied in disparate literatures, hindering the understanding of how these dynamic and intertwined developmental processes unfold. The current study aimed at investigating the concurrent and longitudinal links between the exploration of, and commitment to personal, national, and ethnic identities by immigrant youth. These links were studied separately for exploration and commitment using a cross-lagged panel modelling approach. Three main results emerged. First, robust positive within-time associations among all three identity domains emerged, at all three time points, both for exploration and commitment. Second, there was some evidence for longitudinal negative cross-lagged links between national and ethnic identities for both exploration and commitment, indicating that adolescents who explore and/or commit more to national Greek identity tend to explore and/or commit less to their ethnic identity the following year, and vice versa. However, for the most part, ethnic and/or national identity processes were not longitudinally linked to personal identity processes. Third, most significant cross-lagged effects happened in the first time-window, between T1 and T2. Sensitivity multigroup analyses based on immigrant generation (first vs second generation) and ethnicity (Albanian, Pontian Greek, Others) largely supported these results, notwithstanding the small sizes of some of these sub-samples. These findings contribute to the growing literature on the interplay of personal and social identity development among immigrant and ethnically and racially diverse youth. Further, these findings need to be considered in light of the developmental stage of this study (early adolescence), as well as in light of the broader societal context in contemporary Greece, which is a highly assimilatory society (Pavlopoulos & Motti-Stefanidi, 2017).

### Concurrent Links between Immigrant Youth’s Personal, Ethnic, and National Identities

For both exploration and commitment, positive concurrent correlations between personal, ethnic, and national identities were found for all time points. That is, immigrant adolescents who engaged in relatively greater exploration in one of these identity domains, also tended to engage in relatively greater exploration in the other two domains. Similarly, immigrant adolescents who endorsed relatively higher commitment to one of these identity domains, also tended to report relatively stronger commitments to the other two domains.

This pattern of positive concurrent correlations indicates that these identity domains are part of a broader self-system (Oyserman et al., 2012). The documented positive correlated change supports the idea that ethnic, national and personal identities form a ‘base-layer’ upon which the quest for identity is built. Recent meta-analytic evidence also suggests that exploration and commitment are universal aspects of identity, not necessarily tapping specific domains (Yip et al., 2019). As Yip et al. (2019) note, Erikson’s ideas (1968) were not specific to ‘personal’, or ‘ethnic’, or ‘national’ identities, but rather more generic about exploring and knowing who one is. The results of the current study are in line with the argument that forming an identity, particularly in the case of immigrant youth, involves exploration and commitment with respect to aspects of the self, such as roles, values, beliefs, and lifestyles (Erikson, 1968), as well as with respect to the meaning that one’s ethnic and national background have for the individual (Umaña-Taylor et al., 2014).

### Longitudinal Links between Immigrant Youth’s Personal, Ethnic, and National Identities

Overall, only four of the 24 cross-lagged effects emerged as significant, which is not surprising. Because of the strong autoregressive stability that emerged over time and the robust within-time associations among all three identity domains for both exploration and commitment, there remained little variance to be explained by cross-lagged effects. Furthermore, the relative scarcity of many longitudinal effects paired with the robust and consistent emergence of strong concurrent effects implies that the dynamics among different identity processes and domains might be taking place in shorter timescales than from year-to-year, as recent research on adolescent development shows (Boele et al., 2021). From the significant cross-lagged effects, two general findings are noteworthy.

First, a greater number of cross-lagged associations was documented in the first time window, between T1 and T2, than in the second time window, between T2 and T3. Developmental and contextual approaches might explain this finding. Even though identity work might already have started before early adolescence, it is exactly this period when identity work becomes more salient (Crocetti et al., 2008). Given the emergence of identity work as a developmental task at this stage, a livelier dynamic between processes might be expected. In addition, this study took place during the three years of middle school; during T1 students had just changed schools, having transitioned from primary to secondary education. This broad change in context includes a change in young adolescents’ social environment including new schoolmates and friendships; these changes might have sparked the importance of their immigrant background, and possibly its distinctiveness from the majority group, intensifying, thus, identity dynamics. This way, the identity dynamics during the immediate post-transition phase might be more intense compared to the identity dynamics after they have spent at least a year in the same school environment.

A second general finding was that, in agreement with hypotheses, both in the case of identity exploration and identity commitment, several negative cross-lagged links were observed between national and ethnic identities. Specifically, for the total sample it was found that relatively higher exploration of ethnic identity at T1 predicted relative decreases in exploration of national identity at T2, which in turn predicted further relative increases in exploration of ethnic identity at T3. Furthermore, it was found that relatively higher commitment to ethnic identity at T1 predicted relative decreases in commitment to national identity at T2, and vice versa. Again, developmental and contextual considerations might explain these findings. From a cognitive-developmental perspective (Amiot et al., 2007), early adolescence is a developmental stage when multiple aspects of identity are mostly compartmentalized and context-dependent. Identity integration, the fourth and last stage in the cognitive-developmental model of identity (Amiot et al., 2007), happens later, from middle adolescence onwards. It is possible, therefore, that this negative longitudinal interplay between ethnic and national identities would be weaker or even absent if this study extended to middle adolescence and beyond. Furthermore, these findings reflect the attitudes of more assimilationist receiving societies (Ward & Geeraert, 2016), such as Greece (Pavlopoulos & Motti-Stefanidi, 2017). In assimilationist societies, immigrant people are forced to choose between the national culture and their own culture, resulting in a negative relation between the two (Ward & Geeraert, 2016). In such a societal context, dual identities, which would be reflected in a positive relation between ethnic and national identities, may be contested by the receiving society, as well as by immigrants’ co-ethnic community (Schwartz et al., 2018; Wiley et al., 2019). Thus, in such countries, a strong national orientation and identity may be incompatible with a strong orientation towards, and sense of belonging to, one’s ethnic culture. In fact, recent evidence even from more accepting societies like the Dutch, showed that stronger enactment of dual identity by immigrant youth was associated with stronger identity denial by their peers (Cárdenas et al., 2021). Thus, given the strong context-dependence of identity integration during this developmental stage (Amiot et al., 2007), it stands to reason that immigrant youth experience the identity processes (exploration and commitment) between ethnic and national identity domains as incompatible.

This incompatibility found in the current study might be worrisome. Existing evidence shows that a favourable outcome of identity development (Umaña-Taylor et al., 2014) is identity integration, whereby youth can combine and synthesize varying aspects of their identity, without having to choose some aspects against some others. Therefore, stakeholders and practitioners might need to focus on interventions to promote identity integration, by reducing the incompatibility between ethnic and national identity processes in assimilationist contexts, like the one of the current study.

There was little support regarding the longitudinal links of personal identity with ethnic and national identity. The results differed for the two identity processes (commitment, exploration). Regarding *commitment*, the longitudinal links of personal identity commitment with ethnic and national identity commitment, were expected to be bidirectional. However, this hypothesis received no longitudinal support. In contrast, regarding *exploration*, the examination of the link of personal identity exploration with ethnic and national identity exploration was exploratory and received some support. Thus, overall, fewer cross-lagged effects involving personal identity emerged, compared to cross-lagged effects between ethnic and national identities. Given the aforementioned positive cross-sectional links indicating that exploration and commitment processes in different domains (ethnic, national, personal) take place in parallel, the question that arises is why ethnic and/or national identity processes have little longitudinal influence on personal identity processes, and vice versa?

A potential explanation in what regards exploration is that for immigrant youth, and given the assimilationist Greek context, exploring more what their heritage culture means to them is so demanding, that it might absorb their energy to question friendships and educational choices. In other words, for minority youth in assimilationist contexts, group membership might be more salient and thus more urgently needing addressing and exploring, whereas exploring educational and friendship goals and plans can take a ‘back seat’ to that. An alternative possible explanation is that immigrant youth who have explored their ethnic group membership are more certain who they are and ‘settle’ into their current friends and education and need no longer to explore other education/friend goals.

Regarding personal identity commitment, however, the lack of significant cross-lagged effects from and to either ethnic or national identity commitments implies that, commitment in education and friendships remains relatively sealed from the negative dynamic between national and ethnic commitment. That is, regardless the negatively charged dynamic between ethnic and national identities and their relative standing in these aspects of social identity, immigrant youth can still commit strongly to education and friendships. Given the important implications of personal identity commitment, as conceptualized in this study, for future psychosocial adaptation (Kaniušonytė et al., 2019), this tentative dissociation of personal identity commitment from questions of national and ethnic identity might be an interesting topic to investigate further in future studies.

### Strengths, Limitations, and Future Directions

This study has several strengths that add credibility to the conclusions. First, the longitudinal design of this study allowed a focus on the developmental processes that are at play in immigrant youth identity development. Second, the panel design with several repeated measurements of different domains of identity development allowed a look into the dynamics between the disparate fields of personal, ethnic, and national identity research. Accordingly, based on the present findings, suggestions were offered for advancing theoretical enterprise on identity development across personal and social identity domains to increase a cross-talk between these literatures and generate a more nuanced account of youth’s quest for identity. Third, the sample comes from Greece, a country that receives a large volume immigration in the European Union but tends to be underrepresented in research.

Despite this study’s strengths, several limitations are noteworthy in interpreting the study’s conclusions, which also provide opportunities for future research. First, the results were based solely on self-report measures. Even though self-reports are often a legitimate means of investigating internal, psychological processes, such as identity development, other methods (e.g., observed behaviour) could have enriched the current data and might have provided additional insights. Second, some of the scales in this study showed moderate internal consistency coefficient, which might have affected the results. Third, the conceptualization of personal identity was based on exploration and commitment in the domains of education and friendships. Despite this conceptualization being based on a sound framework and measurement instrument (Crocetti et al., 2008), which has been commonly used in developmental research on identity (Crocetti et al., 2017), investigating dynamic development at multiple timescales (Becht et al., 2021), such a conceptualization might differ from other conceptualizations, such as personal identity as ‘coherence’ and ‘confusion’, in relevant research (Meca et al., 2017). Fourth, this study only used annual assessments. It is possible that behavioural and psychological processes involved in identity development might be taking place at shorter time scales, such as months, weeks, days, or even moment-to-moment interactions between a young person and their social environments. Thus, future studies will be wise to measure identity developmental processes at these different timescales. This approach could offer a more detailed look into the (micro) processes that are at play when youth are in the quest for identity development. Fifth, the present sample was not randomly chosen; instead, schools in urban areas with high immigrant density were oversampled. Therefore, the results presented herein might not represent immigrant youth living in other urban areas less densely populated with immigrants or in rural areas. Future research should also examine identity development processes across multiple contexts as permitted by ecological-momentary sampling. Another promising avenue for future research centres on using person-centred approaches, as opposed to variable-centered ones on which the present study relied, to represent distinct constellations of personal, ethnic, and national identity during adolescence (Cheon et al., 2020).

## Conclusion

Ethnic, national, and personal identity development are essential aspects of immigrant youth’s psychosocial adjustment that have been treated mostly in different research lines. This study aimed at investigating the longitudinal dynamic interconnections among ethnic, national, and personal identity exploration and commitment, with the goal to make a first step to integrating the largely disparate extant literatures on these domains of identity. To achieve this, the current study followed a large sample of immigrant early adolescents living in Greece across middle school. Three main findings emerged. First, positive and strong within-time links between ethnic, national and personal identities were found at all three time points for both exploration and commitment, which suggests that these processes co-occur for all three identity domains, and for both identity processes. Second, there was some evidence showing that ethnic and national identities were negatively linked over time and they seemed largely disconnected from the formation of immigrant youth’s personal identity. These results suggest that in this assimilatory immigrant receiving society dual identities are not often achieved by immigrant youth. A well-formed and rounded identity, that includes integrated aspects of personal and social identities would be expected to promote youth’s current and long-term adaptation and psychological well-being and would benefit both immigrants and the receiving society. Third, most significant cross-lagged effects emerged during early adolescence, which implies that this developmental stage might be particularly important to target for possible supportive interventions. Policy and programme initiatives, which will promote a positive public attitude towards immigrants, are needed. A positive attitude of nonimmigrants is a prerequisite for immigrant youth to develop dual identities which they will be able to integrate into their personal identity.

## Supplementary information

Supplementary Material

## References

[CR1] Aguinis H, Gottfredson RK, Joo H (2013). Best-practice recommendations for defining, identifying, and handling outliers. Organizational Research Methods.

[CR2] Albarello F, Crocetti E, Rubini M (2018). I and us: a longitudinal study on the interplay of personal and social identity in adolescence. Journal of Youth and Adolescence.

[CR3] Amiot CE, de la Sablonnière R, Terry DJ, Smith JR (2007). Integration of social identities in the self: toward a cognitive-developmental model. Personality and Social Psychology Review.

[CR4] Becht AI, Nelemans SA, Branje SJT, Vollebergh WAM, Meeus WHJ (2021). Daily identity dynamics in adolescence shaping identity in emerging adulthood: an 11-year longitudinal study on continuity in development. Journal of Youth and Adolescence.

[CR5] Boele, S., Nelemans, S., Denissen, J., Prinzie, P., Bülow, A., & Keijsers, L. (2021). Testing transactional processes between parental support and adolescent depressive symptoms: from a daily to a biennial timescale. PsyArXiv. 10.31234/osf.io/jzr4v. 10.1017/S0954579422000360 35545300

[CR6] Branje, S. J. T, Mastrotheodoros, S. & Laursen, B. (2021) Family relationships during adolescence. In A. L. Vangelisti (Ed.), *Routledge handbook of family communication* (3rd ed.). Routledge.

[CR7] Cárdenas D, Verkuyten M, Fleischmann F (2021). “You are too ethnic, you are too national”: dual identity denial and dual identification. International Journal of Intercultural Relations.

[CR8] Cheon, Y. M., Ip, P. S., Haskin, M., & Yip, T. (2020). Profiles of adolescent identity at the intersection of ethnic/racial identity, American identity, and subjective social status. *Frontiers in Psychology*, 11. 10.3389/fpsyg.2020.00959. 10.3389/fpsyg.2020.00959 PMC7244255 32499743

[CR9] Cheung GW, Rensvold RB (2002). Evaluating goodness-of-fit indexes for testing measurement invariance. Structural Equation Modeling: A Multidisciplinary Journal.

[CR10] Crocetti E, Branje S, Rubini M, Koot HM, Meeus W (2017). Identity processes and parent-child and sibling relationships in adolescence: a Five-wave Multi-informant Longitudinal Study. Child Development.

[CR11] Crocetti, E., Cieciuch, J., Gao, C. H., Klimstra, T., Lin, C. L., Matos, P. M., Morsünbül, Ü., Negru, O., Sugimura, K., Zimmermann, G., & Meeus, W. (2015). National and gender measurement invariance of the Utrecht-Management of Identity Commitments Scale (U-MICS): a 10-nation study with university students. *Assessment*. 10.1177/1073191115584969. 10.1177/1073191115584969 25944797

[CR12] Crocetti E, Rubini M, Meeus W (2008). Capturing the dynamics of identity formation in various ethnic groups: development and validation of a three-dimensional model. Journal of Adolescence.

[CR13] Dimitrova R, Buzea C, Taušová J, Uka F, Zakaj S, Crocetti E (2018). Relationships between identity domains and life satisfaction in minority and majority youth in Albania, Bulgaria, Czech Republic, Kosovo, and Romania. European Journal of Developmental Psychology.

[CR14] Erikson E (1968). Youth: identity and crisis.

[CR15] Jorgensen, T. D., Pornprasertmanit, S., Schoemann, A. M., & Rosseel, Y. (2020). *semTools: useful tools for structural equation modeling*. https://cran.r-project.org/package=semTools.

[CR17] Jugert P, Pink S, Fleischmann F, Leszczensky L (2020). Changes in Turkish- and resettler-origin adolescents’ acculturation profiles of identification: a Three-year Longitudinal Study from Germany. Journal of Youth and Adolescence.

[CR18] Jugert P, Šerek J, Stollberg J (2019). Contextual moderators of the link between national and European identity among European youth. Journal of Youth Studies.

[CR19] Kaniušonytė G, Truskauskaitė‐Kunevičienė I, Žukauskienė R, Crocetti E (2019). Knowing who you are for not feeling lonely? A longitudinal study on identity and loneliness. Child Development.

[CR20] Kroger, J., & Marcia, J. E. (2011) The identity statuses: origins, meanings, and interpretations. In S. J. Schwartz, K. Luyckx, & V. L. Vignoles (Eds.), *Handbook of identity theory and research* (pp. 31–53). New York: Springer. 10.1007/978-1-4419-7988-9_2.

[CR22] Martinez-Fuentes S, Umaña-Taylor AJ, Jager J, Seaton EK, Sladek MR (2020). An examination of ethnic-racial identity and U.S. American identity among Black, Latino, and White adolescents. Identity.

[CR24] Mastrotheodoros S, Pavlopoulos V, Motti-Stefanidi F (2021). Utrecht-Management of Identity Commitments Scale (UMICS): Greek adaptation and measurement invariance across time and ethnic groups. European Journal of Developmental Psychology.

[CR25] Mastrotheodoros S, Van der Graaff J, Deković M, Meeus WHJ, Branje SJT (2019). Coming closer in adolescence: convergence in mother, father, and adolescent reports of parenting. Journal of Research on Adolescence.

[CR26] Meca, A., Sabet, R. F., Farrelly, C. M., Benitez, C. G., Schwartz, S. J., Gonzales-Backen, M., Lorenzo-Blanco, E. I., Unger, J. B., Baezconde-Garbanati, L., Picariello, S., Soto, D. W., Pattarroyo, M., Des Rosiers, S. E., Villamar, J. A., & Lizzi, K. M. (2017). Personal and cultural identity development in recently immigrated hispanic adolescents: links with psychosocial functioning. *Cultural Diversity and Ethnic Minority Psychology*. 10.1037/cdp0000129. 10.1037/cdp0000129 PMC5491363 28206778

[CR27] Motti-Stefanidi F, Crockett LJ, Carlo G, Schulenberg J (2021). Immigrant youth resilience in the context of challenging receiving societies. APA handbook of adolescent and young adult development.

[CR28] Motti-Stefanidi F, Asendorpf JB (2017). Adaptation during a great economic recession: a cohort study of Greek and immigrant youth. Child Development.

[CR29] Motti-Stefanidi, F., Berry, J., Chryssochoou, X., Sam, D. L., & Phinney, J. (2012). Positive immigrant youth adaptation in context: developmental, acculturation, and social-psychological perspectives. In A. S. Masten, K. Liebkind, & D. J. Hernandez (Eds.), *Realizing the potential of immigrant youth*. (pp. 117–158). Cambridge University Press. 10.1017/CBO9781139094696.008.

[CR30] Nguyen A-MD, Benet-Martínez V (2013). Biculturalism and adjustment: a meta-analysis. Journal of Cross-Cultural Psychology.

[CR31] OECD. (2016). *International migration outlook 2016*. https://read.oecd-ilibrary.org/social-issues-migration-health/international-migration-outlook-2016_migr_outlook-2016-en.

[CR32] OECD, & European Commission. (2015). *Indicators of immigrant integration 2015*. https://read.oecd-ilibrary.org/social-issues-migration-health/indicators-of-immigrant-integration-2015-settling-in_9789264234024-en.

[CR33] Oyserman, D., Elmore, K., & Smith, G. (2012). Self, self-concept, and identity. In M. R. Leary, & J. P. Tangney (Eds.), *Handbook of self and identity*. Guilford Press.

[CR34] Pavlopoulos, V., & Motti-Stefanidi, F. (2017). Intercultural relations in Greece. In J. W. Berry (Ed.), *Mutual intercultural relations* (pp. 187–209). Cambridge University Press. 10.1017/9781316875032.

[CR35] Phinney JS, Horenczyk G, Liebkind K, Vedder P (2001). Ethnic identity, immigration, and well-being: an interactional perspective. Journal of Social Issues.

[CR36] Phinney, J. S., & Ong, A. D. (2007). Conceptualization and measurement of ethnic identity: current status and future directions. *Journal of Counseling Psychology*. 10.1037/0022-0167.54.3.271.

[CR37] Schwartz, S. J., Luyckx, K., & Vignoles, V. (2011). In S. J. Schwartz, K. Luyckx, & V. L. Vignoles (Eds.), *Handbook of identity theory and research*. Springer. 10.1007/978-1-4419-7988-9.

[CR38] Schwartz SJ, Meca A, Ángel Cano M, Lorenzo-Blanco EI, Unger JB (2018). Identity development in immigrant youth. European Psychologist.

[CR39] Schwartz SJ, Zamboanga BL, Weisskirch RS, Rodriguez L (2009). The relationships of personal and ethnic identity exploration to indices of adaptive and maladaptive psychosocial functioning. International Journal of Behavioral Development.

[CR40] Spears, R. (2011) Group identities: the social identity perspective. In S. J. Schwartz, V. Vignoles, & K. Luyckx (Eds.), *Handbook of identity theory and research* (pp. 201–224). New York: Springer. 10.1007/978-1-4419-7988-9_9.

[CR42] Syed M, McLean KC (2016). Understanding identity integration: theoretical, methodological, and applied issues. Journal of Adolescence..

[CR43] Tajfel, H., & Turner, J. C. (2004). The social identity theory of intergroup behavior. In J. T. Lost, & J. Sidanius (Eds.), *Political psychology* (pp. 276–293). Psychology Press. 10.4324/9780203505984-16.

[CR44] Tierney, N. J., & Cook, D. H. (2018). Expanding tidy data principles to facilitate missing data exploration, visualization and assessment of imputations. https://arxiv.org/abs/1809.02264.

[CR45] Triandis HC (2001). Individualism-collectivism and personality. Journal of Personality.

[CR46] Umaña-Taylor, A. J. (2011) Ethnic identity. In S. J. Schwartz, V. Vignoles, & K. Luyckx (Eds.), *Handbook of identity theory and research* (pp. 791–809). New York: Springer. 10.1007/978-1-4419-7988-9_33.

[CR47] Umaña-Taylor AJ, Kornienko O, McDermott ER, Motti-Stefanidi F (2020). National identity development and friendship network dynamics among immigrant and non-immigrant youth. Journal of Youth and Adolescence.

[CR48] Umaña-Taylor AJ, Quintana SM, Lee RM, Cross WE, Rivas-Drake D, Schwartz SJ, Syed M, Yip T, Seaton E (2014). Ethnic and racial identity during adolescence and into young adulthood: an integrated conceptualization. Child Development.

[CR49] van Buuren SV, Groothuis-Oudshoorn K (2011). Mice: multivariate imputation by chained equations in R. Journal of Statistical Software.

[CR50] Verkuyten, M., Wiley, S., Deaux, K., & Fleischmann, F. (2019). To be both (and More): immigration and identity multiplicity. *Journal of Social Issues*. 10.1111/josi.12324.

[CR51] Ward C, Geeraert N (2016). Advancing acculturation theory and research: the acculturation process in its ecological context. Current Opinion in Psychology.

[CR53] Wiley S, Fleischmann F, Deaux K, Verkuyten M (2019). Why immigrants’ multiple identities matter: implications for research, policy, and practice. Journal of Social Issues.

[CR54] Wu H, Estabrook R (2016). Identification of confirmatory factor analysis models of different levels of invariance for ordered categorical outcomes. Psychometrika.

[CR55] Yip T, Wang Y, Mootoo C, Mirpuri S (2019). Moderating the association between discrimination and adjustment: a meta-analysis of ethnic/racial identity. Developmental Psychology.

