# Supplementary material for: Developmental Interplay between Ethnic, National, and Personal Identity in Immigrant Adolescents
Source: J Youth Adolesc. 2021 Apr 17;50(6):1126–39. doi: 10.1007/s10964-021-01434-y (PMC8116219; doi:10.1007/s10964-021-01434-y)
Supplement: Supplementary file 1 — Supplementary Material [file 10964_2021_1434_MOESM1_ESM.docx]

Supplementary Material

Results of multigroup analyses based on immigrant generation and ethnicity (2 tables and 4 figures).

Table S1

T-test comparisons between cases participating in all three waves and those who missed at least one wave.

|  | | | | | | | | | |
| --- | --- | --- | --- | --- | --- | --- | --- | --- | --- |
|  | | **t** | | **df** | | **p** | | **Cohen's d** | |
| gender_1 |  | 1.571 |  | 721 |  | 0.117 | ᵃ | 0.134 |  |
| ethncty_1 |  | -0.364 |  | 721 |  | 0.716 | ᵃ | -0.031 |  |
| adv_1 |  | -1.146 |  | 622 |  | 0.252 |  | -0.113 |  |
| adv_2 |  | -3.231 |  | 600 |  | 0.001 |  | -0.355 |  |
| adv_3 |  | -2.206 |  | 537 |  | 0.028 |  | -0.331 |  |
| meimexpl_1 |  | 0.039 |  | 595 |  | 0.969 |  | 0.004 |  |
| meimexpl_2 |  | 0.116 |  | 566 |  | 0.908 |  | 0.013 |  |
| meimexpl_3 |  | 0.536 |  | 515 |  | 0.592 |  | 0.085 |  |
| meimcom_1 |  | -0.976 |  | 595 |  | 0.330 |  | -0.098 |  |
| meimcom_2 |  | 0.152 |  | 566 |  | 0.880 |  | 0.017 |  |
| meimcom_3 |  | -0.218 |  | 515 |  | 0.827 |  | -0.034 |  |
| nidexpl_1 |  | 1.192 |  | 619 |  | 0.234 | ᵃ | 0.118 |  |
| nidexpl_2 |  | 0.304 |  | 596 |  | 0.761 |  | 0.034 |  |
| nidexpl_3 |  | -0.007 |  | 532 |  | 0.994 |  | -0.001 |  |
| nidcom_1 |  | 0.705 |  | 618 |  | 0.481 |  | 0.070 |  |
| nidcom_2 |  | 1.244 |  | 596 |  | 0.214 |  | 0.137 |  |
| nidcom_3 |  | -0.557 |  | 532 |  | 0.578 |  | -0.085 |  |
| com_1 |  | 0.112 |  | 622 |  | 0.911 |  | 0.011 |  |
| com_2 |  | 0.532 |  | 600 |  | 0.595 |  | 0.058 |  |
| com_3 |  | 1.588 |  | 535 |  | 0.113 | ᵃ | 0.240 |  |
| exp_1 |  | 0.129 |  | 622 |  | 0.897 |  | 0.013 |  |
| exp_2 |  | 1.233 |  | 600 |  | 0.218 |  | 0.136 |  |
| exp_3 |  | 2.192 |  | 535 |  | 0.029 |  | 0.332 |  |
| age_1 |  | -8.112 |  | 622 |  | < .001 | ᵃ | -0.814 |  |
|  | | | | | | | | | |
| *Note:* ethncty: ethnicity; adv: socioeconomic adversity; meimexpl: ethnic identity exploration; meimcom: ethnic identity commitment; nidexpl: national identity exploration; nidcom: national identity commitment; com: personal identity commitment; expl: personal identity exploration.  ᵃ Levene's test is significant (p < .05), suggesting a violation of the equal variance assumption | | | | | | | | | |
|  | | | | | | | | | |

Figure S1. Multigroup Model Based on Immigrant Generation: Standardized Coefficients for the Cross-Lagged Effects Among Personal Identity Exploration, National Identity Exploration, and Ethnic Identity Exploration. Coefficients for 1^st^ generation immigrants are shown on the left of the slash; for 2^nd^ generation immigrants on the right.

Note: Adolescent sex, ethnicity, and family SES were controlled for. Results are pooled over 20 multiply imputed data sets, generated over 10 iterations each.

N_1st_ _generation_ = 152, N_2nd generation_ = 571. CFI = 1.00; TLI = 1.97; RMSEA = .000; x^2^ = 199, df = 834.

Figure S2. Multigroup Model Based on Immigrant Generation: Standardized Coefficients for the Cross-Lagged Effects Among Personal Identity Commitment, National Identity Commitment, and Ethnic Identity Commitment. Coefficients for 1^st^ generation immigrants are shown on the left of the slash; for 2^nd^ generation immigrants on the right.

Note: Adolescent sex, ethnicity, and family SES were controlled for. Results are pooled over 20 multiply imputed data sets, generated over 10 iterations each.

N_1st_ _generation_ = 152, N_2nd generation_ = 571. CFI = 1.00; TLI = 1.07; RMSEA = .000; x^2^ = 189, df = 441.

Figure S3. Multigroup Model Based on Ethnicity: Standardized Coefficients for the Cross-Lagged Effects Among Personal Identity Exploration, National Identity Exploration, and Ethnic Identity Exploration. Coefficients represent Albanian/Pontic-Greek/Other ethnicities.

Note: Adolescent sex, immigrant generation, and family SES were controlled for. Results are pooled over 20 multiply imputed data sets, generated over 10 iterations each.

N_Albanian_ = 362, N_Pontic Greek_  = 138, N_Other_ = 223, CFI = 1.00; TLI = 1.17; RMSEA = .000; x^2^ = 272, df = 1251.

Figure S4. Multigroup Model Based on Ethnicity: Standardized Coefficients for the Cross-Lagged Effects Among Personal Identity Commitment, National Identity Commitment, and Ethnic Identity Commitment. Coefficients represent Albanian/Pontic-Greek/Other ethnicities.

Note: Adolescent sex, immigrant generation, and family SES were controlled for. Results are pooled over 20 multiply imputed data sets, generated over 10 iterations each.

N_Albanian_ = 362, N_Pontic Greek_  = 138, N_Other_ = 223, CFI = 1.00; TLI = 1.60; RMSEA = .000; x^2^ = 302, df = 1251.

Table S2

Fit indices for the three-factor model of the U-MICS for each domain and each year, for measurement invariance across time points for each group and each domain.

|  | χ² | *df* | CFI | TLI | RMSEA[90%CI] | Models Compared | ΔCFI | ΔRMSEA |
| --- | --- | --- | --- | --- | --- | --- | --- | --- |
| Longitudinal Measurement Invariance | | | | | | | | |
| Greek | | | | | | | | |
| *Education***** | | | | | | | | |
| 1. Configural | 514 | 519 | 1.00 | 1.00 | 0.000[.000-.015] |  |  |  |
| 2. Thresholds | 552 | 567 | 1.00 | 1.01 | 0.000[.000-.013] | M2-M1 | .000 | .000 |
| 3. Loadings | 569 | 585 | 1.00 | 1.01 | 0.000[.000-.013] | M3-M2 | .000 | .000 |
| 4. Intercepts | 514 | 519 | 1.00 | 1.00 | 0.000[.000-.015] | M4-M3 | .000 | .000 |
| *Interpersonal* | | | | | | | | |
| 1. Configural | 654 | 624 | .994 | 0.993 | 0.011[.000-.019] |  |  |  |
| 2. Thresholds | 688 | 676 | .998 | 0.997 | 0.006[.000-.016] | M2-M1 | .004 | -.005 |
| 3. Loadings | 715 | 696 | .996 | 0.996 | 0.008[.000-.017] | M3-M2 | -.002 | .002 |
| 4. Intercepts | 753 | 716 | .993 | 0.992 | 0.011[.000-.019] | M4-M3 | -.003 | .003 |
| Albanian | | | | | | | | |
| *Education***** | | | | | | | | |
| 1. Configural | 402 | 519 | 1.00 | 1.12 | 0.000[.000-.000] |  |  |  |
| 2. Thresholds | 441 | 567 | 1.00 | 1.12 | 0.000[.000-.000] | M2-M1 | .000 | .000 |
| 3. Loadings | 473 | 585 | 1.00 | 1.10 | 0.000[.000-.000] | M3-M2 | .000 | .000 |
| 4. Intercepts | 525 | 603 | 1.00 | 1.07 | 0.000[.000-.000] | M4-M3 | .000 | .000 |
| *Interpersonal* | | | | | | | | |
| 1. Configural | 623 | 624 | 1.00 | 1.00 | 0.000[.000-.016] |  |  |  |
| 2. Thresholds | 676 | 676 | 1.00 | 1.00 | 0.000[.000-.016] | M2-M1 | .000 | .000 |
| 3. Loadings | 712 | 696 | .992 | 0.992 | 0.008[.000-.018] | M3-M2 | -.008 | .008 |
| 4. Intercepts | 697 | 716 | 1.00 | 1.01 | 0.000[.000-.013] | M4-M3 | .009 | -.008 |

Note. Items were specified as ordinal variables and the DWLS estimator with robust standard errors was used.

**** Item 11 from the education domain (translated in Greek as ‘*I often think it would be better to learn different things at school’*) was excluded at all three waves, for both Greek and Albanian longitudinal measurement invariance models, due to several cross-loadings.
